# Intraoperative application of a new-generation 3D IV-DSA technology in resection of a hemorrhagic cerebellar AVM

**DOI:** 10.3171/2020.10.FOCVID2086

**Published:** 2021-01-01

**Authors:** Burak Ozaydin, Demi W. Dawkins, Stephanie A. Armstrong, Beverly Aagaard-Kienitz, Mustafa K. Baskaya

**Affiliations:** Departments of 1 Neurological Surgery and; 2 Radiology, University of Wisconsin School of Medicine and Public Health, Madison, Wisconsin

**Keywords:** arteriovenous malformation, cone-beam CT, intraoperative angiography, intravenous digital subtraction angiography, IV-DSA, posterior fossa

## Abstract

Although intravenous digital subtraction angiography (IV-DSA), cone-beam CT, and rotational angiography are well-established technologies, using them in a single system in the hybrid operating room to acquire high-quality noninvasive 3D images is a recent development. This video demonstrates microsurgical excision of a ruptured cerebellar arteriovenous malformation (AVM) in a 66-year-old male followed by intraoperative IV-DSA acquisition using a new-generation system (Artis Icono). IV-DSA confirmed in real time that no residual remained following excision without the need to reposition the patient. To the best of the authors’ knowledge, this is the first surgical video to demonstrate the simplified workflow and application of this technology in neurovascular surgery.

The video can be found here: https://youtu.be/bo5ya9DQQPw

## Transcript

**0:30 Introduction.** This surgical video demonstrates microsurgical excision of a hemorrhagic right cerebellar arteriovenous malformation in the hybrid operating room/angiography suite. We utilized intraoperative intravenous 3D digital subtraction angiography to confirm in real time that no residual remained following excision. To the best of our knowledge, this is the first surgical video to demonstrate the use of this technology in a real-life use scenario.

**0:46 Background Information.** Although intravenous digital subtraction angiography, cone-beam 3D CT, and intraoperative rotational angiography are well-established technologies, using the combination of these three techniques in a single system in the hybrid operating room to acquire high-quality noninvasive 3D images is a recent development.^1–6^

The system that we used, in this case, is Artis Icono, which is a new-generation angiography system. This system includes novel acquisition geometries focused on minimizing artifacts associated with cone-beam CT, including a sine geometry algorithm used for high-quality cone-beam CT and 3D digital subtraction angiography imaging obtained with intravenous contrast injection. The system also allows highly flexible movement of the C-arm, allowing a wider range of surgical positions and easier transitions between surgical and imaging positions. This advancement enables smoother workflow during intraoperative image acquisition by having stored positions for both imaging and surgical setups.

IV-DSA has made several advancements over the years, and in patients where arterial access is contraindicated or in the hybrid OR setting where rapid and convenient imaging is critical, it is a viable option. This imaging modality has a higher temporal and spatial resolution as well as improved metal artifact correction in comparison with multidetector CT angiography or MR angiography. With the advances made in both imaging quality and hybrid OR workflow with the Artis Icono system, we believe that IV 3D DSA is a critical tool in the arsenal for intraoperative management of complex neurovascular pathologies.

**2:25 Case Presentation.** The patient, in this case, was a 66-year-old male with a history of a known cerebellar AVM, who presented with a new-onset headache, which had started a week prior. Over the few days prior to admission, he had multiple falls characterized by a sensation of dizziness and loss of balance. On the day of admission, he was found down, responsive but nonsensical by his family, and was admitted for emergency care and diagnostic workup.

The initial neurological assessment showed disorientation to time but was otherwise unremarkable.

Initial CT revealed an acute cerebellar vermian hemorrhage with intraventricular extension and mild fourth ventricular compression along with scattered subarachnoid blood. MRI and MR angiography showed surrounding vasogenic edema and confirmed the presence of a hemorrhagic right cerebellar arteriovenous malformation.

Intraarterial digital subtraction angiography affirmed a Spetzler-Martin grade I right-sided cerebellar arteriovenous malformation with arterial supply from the right posterior inferior cerebellar artery and the right SCA, as well as extracranial supply from the right posterior meningeal artery, the right middle meningeal artery, and the right occipital artery. Early venous drainage was superficial via the transverse sinus through the tentorial veins.

After describing the findings and discussing the viable management options with the patient and the family, upon the patient’s and the family’s agreement, we elected to perform microsurgical excision of this arteriovenous malformation and hematoma evacuation with intraoperative intravenous digital subtraction angiography in the hybrid operating room.

**3:54 Preparation for Surgery.** The patient was positioned prone in the radiolucent head holder. The incision and trajectory of the transverse sinus were marked with the aid of intraoperative neuronavigation. After sterile draping, we made a standard midline linear suboccipital skin incision from above the inion down to the C2. Pericranial graft for use in duraplasty was harvested from the superior margin of the incision, rostral to the inion.

**4:07 Demonstration of Opening.** After defining the transverse and superior sagittal sinus’s boundaries over the bone using the neuronavigation, we performed a suboccipital craniotomy, crossing the transverse sinus for better visualization and easier retraction of the dura and tentorium.

The dura was then opened taking care not to injure the occipital sinus inadvertently and leaving the arachnoid intact. Once the occipital sinus was clearly defined, it was coagulated and divided.

**4:43 AVM Excision.** After the reflection and retraction of the dura, we commenced the microscopic part of the case. We incised the arachnoid of the cisterna magna, which allowed the release of the cerebrospinal fluid.

Our initial inspection revealed multiple dysplastic-looking vessels and arterialized veins over the right cerebellar hemisphere. We began arachnoid microdissection at the tentorial-cerebellar interface to better understand the pathological anatomy and reveal the tentorial bridging veins draining the AVM.

We placed a clip on an arterial feeder emerging from an en passage artery to protect the perfusion of the en passage artery’s vascular territory distal to the AVM.

We then initiated a circumferential corticectomy on the right cerebellar hemisphere encircling the nidus. As we dissected, we identified and divided all the afferent and efferent vascular structures of the malformation. During the dissection, we encountered the vermian hematoma on the medial side of our dissection plane. The hematoma was then evacuated. As we deepened our dissection plane circumferentially, we continued to devascularize the nidus by dividing all the arterial feeders as they were encountered.

We were able to identify a couple of small bridging veins and a large one, which was arterialized and appeared to be the draining vein of the malformation. These were skeletonized along with the identified arterial feeders.

After achieving sufficient dissection on the vermian and tentorial sides, while leaving only the tentorial draining veins intact, we then turned our attention to the inferior margin of the nidus. Starting from the surface and gradually deepening the dissection, we connected all the aspects of the dissection plane in order to disconnect all the feeding vessels from the nidus. Again, we left the arterialized draining vein intact at this point.

After isolating the AVM from its arterial supply and confirming that the draining veins started to turn purple, we began dividing these veins one by one, starting from the superficial draining vein. We then coagulated and cut the tentorial bridging vein drainers and leaving a clip on the largest one.

Now, the AVM was completely devascularized, having no afferent and efferent vessels, we finalized our excision by cutting the remaining attachments of the nidus from the viable cerebellum and removing the malformation en bloc. This was then followed by meticulous hemostasis and placing hemostatic matrix over the resection cavity.

**7:23 IV-DSA Acquisition.** Upon completion of the AVM excision, we began intravenous digital subtraction angiography acquisition. The patient was maintained in the prone position with the wound still open. Sterile drapes were used to cover the surgical site. Through the existing large-bore IV in the right arm, intravenous infusion of 100 ml of contrast followed by a saline flush was performed. 3D imaging was acquired in the arterial phase to complete the intravenous DSA acquisition. Images were processed into 3D DSA and cone-beam CT angiography images and analyzed in real time by our neuroendovascular colleague. Diagnostic images revealed no residual of this excised grade I right cerebellar AVM.

This intraoperative acquisition allowed us to close the craniotomy and incision with confidence that the AVM had been completely removed prior to the formal intraarterial selective catheter-based angiography, which was used as a confirmatory diagnostic study.

**8:18 Closure.** The dural incision was closed with duraplasty by using the previously harvested pericranial graft, and the craniectomy defect was covered with titanium mesh affixed to the bone with 4-mm screws. Muscle, fascia, and subcutaneous tissue were approximated, and the skin was then closed.

**8:35 Intraarterial DSA Acquisition.** After placing the wound dressing and concluding the case, the patient was then flipped back to the supine position, and before the cessation of the general anesthesia, a formal intraarterial selective angiogram was done. This confirmed the complete excision of the AVM without evidence of residual with comparable imaging quality to the intravenous digital subtraction angiography acquisition.

**8:57 Postoperative Course.** The patient woke up without any neurological deficits, and the postoperative course was uneventful. He was initially discharged to rehabilitation and continues to do well without any issues in the postoperative 5-month period.

**9:05 Conclusion.** In conclusion, we would like to discuss a few points regarding intraoperative confirmation of the complete excision of arteriovenous malformations by utilizing 3D IV-DSA.

Even though 3D IV-DSA has made significant advancements over the years, there are some inherent limitations of this modality.

Since the system does not generate dynamic images, visualization of early draining veins may not be possible. For that reason, identification of the retained early venous return caused by a residual nidus is solely based on anatomical identification of these vessels and comparison with preoperative DSA.^7^

It is still early to completely rely on this modality and defer selective intraarterial angiography without conducting large-volume studies to compare the accuracy of the imaging evaluation of the excision between 3D IV-DSA and intraarterial DSA.

Nevertheless, this modality provides rapid and convenient intraoperative image acquisitions without needing to reposition the patient or obtain arterial access, which is particularly valuable in cases performed in the prone position. Thus, peace-of-mind closure is possible with the assurance of knowing that the probability of any residual is unlikely prior to having gold-standard confirmation of complete excision.

## Author Contributions

Primary surgeon: Baskaya. Editing and drafting the video and abstract: Ozaydin, Dawkins, Armstrong. Critically revising the work: Baskaya, Dawkins. Reviewed submitted version of the work: Baskaya, Ozaydin, Dawkins, Armstrong. Approved the final version of the work on behalf of all authors: Baskaya. Supervision: Baskaya. Recorded the footage including the macroscopic portions: Armstrong. Performed all imaging: Aagaard-Kienitz.
